# Coordinating Expression of RNA Binding Proteins with Their mRNA Targets

**DOI:** 10.1038/srep07175

**Published:** 2014-11-24

**Authors:** Huifeng Jiang, Lin Xu, Zhe Wang, Jack Keene, Zhenglong Gu

**Affiliations:** 1 Key Laboratory of Systems Microbial Biotechnology, Tianjin Institute of Industrial Biotechnology, Chinese Academy of Sciences, Tianjin, China; 2 Division of Nutritional Sciences, Cornell University, Ithaca, New York 14853, USA; 3 Department of Molecular Genetics and Microbiology, Duke University Medical Center, Durham, NC 27701, USA

## Abstract

Post-transcriptional regulation by RNA binding proteins (RBPs) plays prominent roles in a variety of biological processes. In this study, by analyzing the global regulatory relationship between RBPs and their target mRNAs in yeast, we discovered that most RBP genes are co-regulated with their target genes, but the RBPs tend to dampen expression variation among their target mRNAs. We further examined a well-studied RBP gene, *PUF3*, and found that the protein decreases the variation of its target mRNAs by differentially affecting their decay. We also constructed a mathematical model to explain the relationship between RBPs and the expression of their target genes. Our results provided new insights into the functional importance of RBPs in coordinating the expression of their target genes.

Coordinating the regulation of functionally related genes by reducing their expression variation in a cell is crucial for the survival of organisms with limited resource in nature^1^,^2^,^3^,^4^. In prokaryotes, expression of functionally related genes can be coordinated by forming operons to reduce their expression fluctuation^5^. It was proposed that posttranscriptional regulons in eukaryotes may play a similar role in coordinating the expression of their target genes during posttranscriptional regulation^6^,^7^,^8^. Gene expression is controlled at multiple steps. Post-transcriptional regulation is mediated by small RNAs (e.g. microRNAs) or RNA binding proteins (RBPs) which usually bind to elements in the 3′ UTR and orchestrate the fate of their targeted mRNAs. The systems properties and evolution of microRNA posttranscriptional regulons have been well studied^9^,^10^,^11^,^12^. A theoretical model for microRNA predicts that they can both modify the mean of their target genes expression level and reduce variance of expression for their target genes^8^. While the functional importance of post-transcriptional regulons becomes increasingly clear, their system properties and evolution remain under-investigated.

RBPs and their regulation have been shown to be important in splicing, stability, translocation and translation of their target mRNAs^3^,^13^. Experimental studies indicated that the function of RBPs on gene expression is complicated and sometimes will be opposite in different growth conditions^14^,^15^,^16^,^17^. Many high-throughput approaches to study RBPs have been recently developed^18^,^19^,^20^. In humans, numerous diseases have been linked to defects in RBP functions^16^,^21^,^22^,^23^,^24^. With many examples of RBPs being identified, it becomes feasible to test whether these post-transcriptional regulons can coordinate the expression of their target genes at the genome level. Budding yeast is an ideal model organism to address this issue, in part, because of the absence of microRNA system in this lineage during evolution^25^. The global identification of target mRNAs for multiple RBPs^26^,^27^ in budding yeast also offers a unique opportunity to examine the regulatory relationships between RBPs and their target mRNAs^28^,^29^.

In this study, we firstly examined the regulatory relationship between RBPs and their target genes in budding yeast using RNA-seq data under different conditions. The relationships between gene expression of each RBP and the mean and variation of its target genes were investigated. We also used a well-studied RBP gene, *PUF3*, as an example to investigate the impact of RBP on the expression of their target genes. Our results indicate that most RBPs are co-regulated with their target genes. Furthermore, they play an important role in coordinating expression variation of their target genes.

## Results

### RBP buffering the expression of target genes

To investigate the general relationship between RBPs and their target genes at transcriptional regulation level, we examined the expression correlation coefficient between RBPs and their targets. We selected 33 RBPs each of which has more than 10 target RNAs in *S. cerevisiae*^27^. The expression of the studied RBPs and their target mRNAs was assessed using currently available RNA-Seq data from 148 expression profiles under multiple conditions in yeast. We calculated the correlation coefficients between the expression level of each RBP with the average expression of all of its mRNA target genes in each condition. The same calculation was also conducted between the level of RBP expression and CD (CD: Coefficient of Deviation, the standard deviation of all target genes expression divided by their mean) of its mRNA target genes.

**Figure 1:**
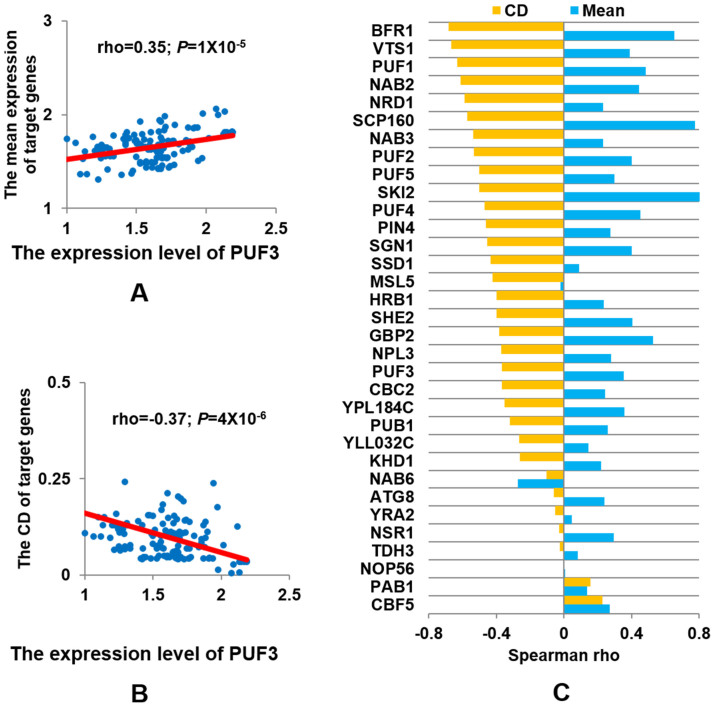
The regulatory relationship for the studied RBPs and their target genes. (A) Positive correlation between *PUF3* gene expression and the mean expression of all its target genes (long_10_RPKM for each gene is used). (B) Negative correlation between *PUF3* expression and the expression variation (CD, *Coefficient* *of Deviation*) of all its target genes. (C) The correlation coefficients (spearman rho) between the gene expression of 33 RBPs and the mean (and CD) of the expression of their target genes. RBPs are ordered based on the spearman rho for the CD values (P-values, for the regression analysis, most of which are statistically significant, were listed in Supplementary Table 1).

Taking *PUF3*, a well-studied RBP gene, as an example, we found that expression of this RBP is positively correlated with the average expression of its mRNA targets (Fig. 1A). In contrast, the CD value of the expression of Puf3p target genes progressively decreases as its expression level increases (Fig. 1B). We further investigated the relationship between all RBPs and the expression and variation of their target genes in various conditions. As shown in Fig. 1C and Supplementary Table 1, strikingly, a similar pattern was observed for most of the 33 RBPs. As CD is the ratio of the standard deviation (SD) of gene expression among targets to their mean expression, to delineate the impact of the mean expression level of the mRNA targets on the negative relationship we observed, we investigated the relationship between the expression of these RBPs and the SD of the expression of their target genes. As shown in Supplementary Fig. 1, the results remained overall similar.

### Puf3p reducing the expression variation of its target genes

**Figure 2:**
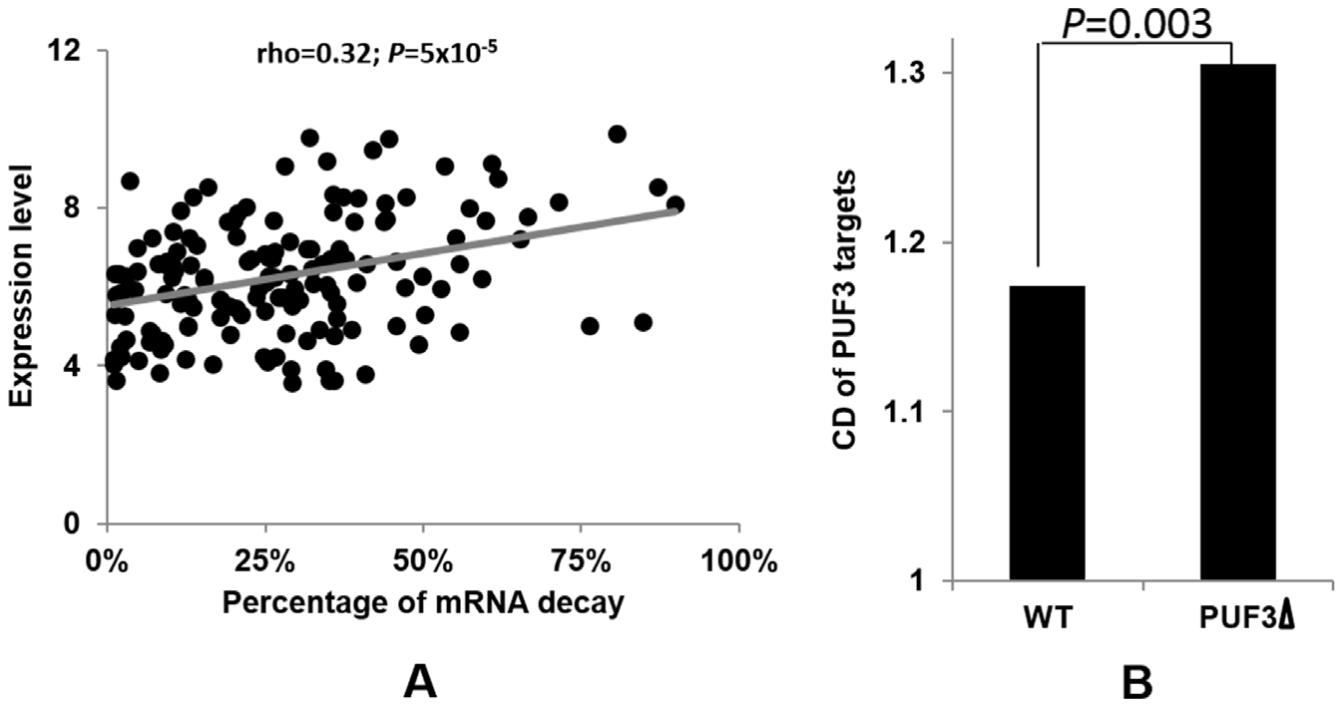
Reducing expression variation of target genes by Puf3p. (A) Biased degradation of Puf3p mRNA targets. The X axis is the percentage of mRNA reduction from the *PUF3Δ* to the WT parental strain, and the Y axis denotes the expression level of Puf3p target genes in the *PUF3Δ* strain (long_2_RPKM for each gene is used). (B) CD values in the WT parental strain and the *PUF3Δ* strain among the Puf3p target genes. F-test was used to test the CD differences.

We used *PUF3* as an example to further investigate the buffering of RBP on the expression of their target genes. The functions of Puf3p and the evolution of this post-transcriptional regulon have been well-established^15^,^26^,^30^,^31^. As Puf3p was shown to be involved in its target mRNA degradation, we investigated the impact of deleting *PUF3* gene on the expression of its targeted mRNA. The expression of the *Puf3p* target genes in the *PUF3* deletion strain was assumed to approximate their expression before *Puf3p* performs its function. The expression of these target genes in the WT strain was assumed to represent their gene expression upon Puf3p function. The genome-wide expression of both strains was profiled using RNA-Seq (Supplementary Figure 2). The degradation efficiency of each Puf3p target gene was defined as the percentage of gene expression level reduced from the *PUF3* deletion mutant to the WT strain. Interestingly, we found that the percentage of mRNA degradation increases with the level of the targeted gene expression (Fig. 2A).

The significantly positive correlation between the degradation efficiency and the original expression level of the target genes indicated that Puf3p may have a selective effect on its target mRNAs based on their level of expression. If this hypothesis is correct, we inferred that this biased mRNA degradation based on the level of gene expression could decrease the gene expression variation among all the Puf3p targeted genes. To test this, we calculated the coefficient of deviation for gene expression among all the Puf3p target genes in the WT parental strain and the *PUF3Δ* strain. As shown in Figure 2B, our results confirmed our prediction and showed that deletion of the *PUF3* gene indeed leads to an increased variation of gene expression among its target genes. (Fig. 2B, F-test *P*-value = 0.003).

### Coordination of gene expression by RBPs and transcription factors (TFs)

**Figure 3:**
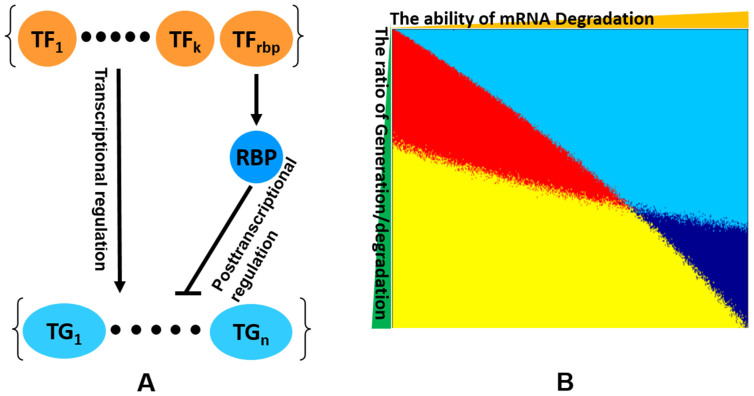
The competition and cooperation between RBPs and TFs. (A) The model of RBPs regulation system was listed. TF_rbp_ activates the expression of RBP and TF_1_ to TF_k_ activate the expression of target genes from TG_1_ to TG_n_. RBP represses the expression of all target genes. (B) The X axis (the parameter j) is the impact of mRNA degradation by RBPs and the Y axis (the parameter m) is the ratio of mRNA generation by TFs and degradation by RBPs. Four colors indicate the relationships between RBPs and the expression of their target genes. The red means a positive correlation with both average and CD of the targets; the yellow means a positive correlation with average and a negative correlation with CD of the targets; the light blue means a negative correlation with the average and a positive correlation with the CD of the targets and the dark blue means a negative correlation with both average and CD of targets.

The observed gene expression results from a joint interaction between generation and degradation of mRNAs. To explain our observation that most RBPs are positively correlated with the average, but negatively correlated with the variation, of the expression of their target genes, we constructed a simple mathematical model to simulate the behavior of RBPs and their targets during gene expression. As shown in Fig. 3A, TFs increase the expression of the target genes and RBPs degrade the transcript of the target genes. According to the relative importance of mRNA generation by TFs and mRNA degradation by RBPs, we could observe four different relationships between RBPs and the expression of their target genes (Fig. 3B). More than 85% studied RBPs are co-expressed with their targets and at the same time reduce the expression variation of the target genes (the yellow region). This relationship between RBPs and their targets occurs when the generation of mRNA by TFs is stronger than the transcript degradation by RBPs, even though the mRNAs of targeted gene are degraded by RBPs. This mode of gene regulation by RBPs is similar to the incoherent feed-forward loop (iFFL) proposed for the miRNA regulation networks where iFFL is frequently used to reduce expression variation of the miRNA target genes^1^,^8^,^32^.

## Discussion

In summary, we discovered that most RBPs tend to be co-regulated with their targeted mRNAs under various conditions, but the up-regulation of RBPs can reduce the expression variation among their targeted mRNAs. According to the combinatorial RNA regulon model, RBPs can be involved in multiple overlapping biological processes^3^. Our results revealed that besides these well-demonstrated functions, many RBPs appear to have an ability to reduce expression variation among their targeted mRNA. Gene expression profiling with a genetic knockout of the yeast *PUF3* gene directly support this conclusion. DNA operons were proposed to enable prokaryotes to synchronize the levels of different components of the same functional pathways, thus reducing the variation that is associated with the production of the functionally related proteins^5^. Upon disappearance of DNA operons in most eukaryotic organisms during evolution, the suppression of expression variation among the targeted mRNAs by RBPs may represent a unique mode to achieve similar functional advantages. We further demonstrated that RBPs could conduct similar functions as miRNAs in other organisms in reducing expression variation. The molecular mechanisms underlying our observation and its evolutionary significance warrant further study, which can also be employed to create coherent biological circuits in genetic engineering^33^,^34^.

## Methods

### RNA-Seq data and the target genes of RBPs

The RBP genes and their targets were downloaded from Dr. Patrick Brown's laboratory^27^. The cutoff to define a target gene for each RBP is q-value < 0.001. Finally 33 RBPs with more than 10 target genes were used in this study. The Fastq files of RNA-Seq data for *S. cerevisiae* were downloaded from the DNAnexus website (http://sra.dnanexus.com/) and NCBI, 148 experimental conditions were listed (Supplementary Table 2). All Fastq files were mapped onto the *S. cerevisiae* genome by SOAP2 and the expression level of each gene was estimated by RPKM (Reads Per Kilobase per Million reads)^35^. After log transformation, the mean, SD and CD of expression for target genes of each RBP were calculated. The correlation coefficients between the expression of RBPs and the mean, SD & CD of their target genes were calculated using cor.test function in R^36^. We also conducted similar analysis using microarray data in different conditions and our conclusion in Fig. 1 still hold true (data not shown).

### Yeast growth condition and RNA Seq experiments

The *PUF3* gene deletion mutant strain (*PUF3Δ*) was from our previous study^31^. Both the *PUF3* gene deletion strain and the wild type BY4741 (MATa, *his3Δ1*, *leu2Δ0, met15Δ0*, *ura3Δ0*) strain were cultured overnight in YPD media. 0.01 OD cells were transferred into fresh YPEG media until 1.0 OD. Total RNA was extracted by the standard Trizol protocol. mRNA was then purified using oligo-dT DynaBeads. cDNA sequencing library was constructed according to the protocol described by Wang et al.^37^. After sequencing, the reads were also mapped into *S. cerevisiae* genome by SOAP2 with no more than two mismatches. RPKM was used to represent the expression level of each gene. The high-throughput sequencing data has been deposited in NCBI Gene Expression Omnibus under the accession number: GSE55419.

### Constructing the gene regulation model

In this model, we assumed that there was a biological system with one transcription factor (TF) and one RNA binding protein (RBP) to regulate 100 target genes (TGs) at the same time. Based on previous studies^38^,^39^, a well-established mathematical model that was used to study transcriptional programs of a gene is: 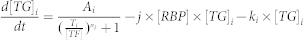


[TG]_i_, [TF] and [RBP] represent the expression level of transcriptional target gene i (i = 1, 2, … 100), transcriptional factor and RNA binding protein at time t, respectively. A_i_ and T_i_ represent the maximal expression level and the activation coefficient for transcriptional target gene i, respectively. In addition, n_i_ and k_i_ are Hill coefficient governing steepness of transcriptional activation term and the decay rate of transcriptional target gene i, respectively. j represents the rate for RBP to break down the TGs, which is dependent on the properties of unique RBP. Different RBPs might have distinct j values. The initial expression level for transcriptional target gene i is defined as [TG]_i0_.

Note that, different transcriptional target gene i might have distinct [TG]_i0_, A_i_, T_i_, n_i_, and k_i_ even when the same TF and RBP regulate them. To compare TFs and RBPs, we assume m = [TF]/[RBP]. As a result, [TF] in the above equation could be substituted by m × [RBP]. In addition, m represents an important parameter to measure the relative importance between TF in producing transcript and RBP in degrading transcripts. Based on a previous experimental estimation for the above parameters in yeast *S. cerevisiae*^40^ and other studies (reviewed in^39^), we assumed the parameter spaces for [TG]_i0_, A_i_, T_i_, n_i_, and k_i_ to be (0, 1), (1, 2), (0, 4), (1, 4) and (0, 1), respectively. Note that, [TG]_i0_ and A_i_ are defined as (0, 1) and (1, 2), which is to avoid the unrealistic situation that [TG]_i0_ could be larger than A_i_ in the random simulation process. Similarly, the parameter space for expression level of RBP ([RBP]) is (0, 2), for which 0 and 2 represent no expression and maximum expression level, respectively. To simply the simulation, we assumed the parameter spaces for m and j to be (0, 2) and (0, 1), respectively. As a result, since m = [TF]/[RBP], (0, 2) as m's parameter space would allow us to simulate both situations that [TF] is higher or lower than [RBP]. Based on all of these reasonable assumptions, we randomly produced a series of parameters for each gene of the 100 TGs. The average expression levels and CD for the 100 TGs under one [RBP] value are calculated. Under each combination of m and j values, this process is repeated 10,000 times with 1,000 different [RBP] values to calculate the correlation coefficients between [RBP] and average expression levels (or CD) of 100 TGs.

## Author Contributions

H.J., J.K. and Z.G. designed experiments. H.J. and L.X. performed data analysis and Z.W. performed the experiments. H.J. and Z.G. wrote the paper. All authors reviewed the manuscript.

## Supplementary Material

Supplementary Information Coordinating Expression of RNA Binding Proteins with Their mRNA Targets
