# Supplementary material for: Coordinating Expression of RNA Binding Proteins with Their mRNA Targets
Source: Sci Rep. 2014 Nov 24;4:7175. doi: 10.1038/srep07175 (PMC4241525; doi:10.1038/srep07175)
Supplement: Supplementary Information — Coordinating Expression of RNA Binding Proteins with Their mRNA Targets [file srep07175-s1.pdf]

Coordinating Expression of RNA Binding Proteins with Their mRNA Targets

Huifeng Jiang[1,2,*], Lin Xu[2], Zhe Wang[2], Jack Keene[3] & Zhenglong Gu[2,*]

[1]Key Laboratory of Systems Microbial Biotechnology, Institute of Tianjin Industrial Biotechnology, Chinese Academy of Sciences, Tianjin, China

[2]Division of Nutritional Sciences, Cornell University, Ithaca, New York 14853, USA

[3]Department of Molecular Genetics and Microbiology, Duke University Medical Center, Durham, NC 27701, USA

Supplementary Table 1-2

Supplementary Figures 1–2

Supplementary Table 1: The regulatory relationship for the studied RBPs and their target genes.

RBPs	*P-Mean* [a]	*rho-Mean* [a]	*P-SD* [a]	*rho-SD* [a]	*P-CD* [a]	*rho-CD* [a]
CBF5	0.0009039	0.2709	3.92E-06	0.3716	0.005846	0.226
PAB1	0.09274	0.1387	0.008289	0.2163	0.05445	0.1584
NOP56	0.9476	0.0054	0.001406	0.2609	0.9568	0.0045
TDH3	0.3151	0.0834	0.4195	0.0671	0.7776	-0.0235
NSR1	0.0002735	0.2959	0.06448	0.1529	0.726	-0.0291
YRA2	0.5675	0.0475	0.3744	0.0738	0.5277	-0.0525
ATG8	0.0038	0.2398	0.000353	0.2937	0.4777	-0.0596
NAB6	0.0008323	-0.2718	0.1411	-0.1215	0.2064	-0.1045
KHD1	0.007009	0.2208	0.004543	-0.232	0.001338	-0.2613
YLL032C	0.08184	0.144	8.00E-06	-0.359	0.001191	-0.2648
PUB1	0.001347	0.2611	0.0004592	-0.2844	7.09E-05	-0.3207
YPL184C	9.65E-06	0.3582	0.0002026	-0.3032	1.38E-05	-0.3522
CBC2	0.00281	0.2453	0.002982	-0.2439	5.71E-06	-0.3668
PUF3	1.19E-05	0.3525	1.59E-05	-0.3477	4.40E-06	-0.3685
NPL3	0.0006563	0.2779	0.0002556	-0.2975	3.67E-06	-0.3726
GBP2	6.83E-12	0.5273	0.0007458	-0.2751	1.76E-06	-0.3824
SHE2	5.67E-07	0.4033	0.009798	-0.2135	8.29E-07	-0.3978
HRB1	0.003687	0.2373	4.30E-08	-0.4317	4.66E-07	-0.4002
MSL5	0.8159	-0.0194	3.77E-05	-0.333	9.78E-08	-0.4225
SSD1	0.2702	0.0915	1.27E-08	-0.4481	4.40E-08	-0.4327
SGN1	4.80E-07	0.4011	3.35E-05	-0.3351	7.60E-09	-0.4541
PIN4	0.0008308	0.2746	2.90E-08	-0.4437	7.40E-09	-0.4606
PUF4	8.14E-09	0.4519	1.33E-08	-0.4461	1.56E-09	-0.4708
SKI2	2.20E-16	0.8104	3.75E-07	0.4097	2.01E-10	-0.5002
PUF5	0.0002383	0.2989	2.20E-16	-0.5416	7.80E-11	-0.5034
PUF2	6.74E-07	0.4008	4.00E-12	-0.5183	2.20E-16	-0.5329
NAB3	0.004614	0.2324	8.09E-15	-0.5841	1.96E-12	-0.5386
SCP160	2.20E-16	0.7753	3.12E-08	-0.4414	2.20E-16	-0.5738
NRD1	0.004833	0.2317	2.20E-16	-0.6756	2.20E-16	-0.5886
NAB2	1.57E-08	0.4454	4.39E-11	-0.5095	2.40E-16	-0.61
PUF1	6.79E-10	0.4828	2.20E-16	-0.6277	2.20E-16	-0.6306
VTS1	1.05E-06	0.3899	2.40E-09	-0.4674	2.20E-16	-0.6675
BFR1	2.20E-16	0.6542	2.20E-16	-0.6437	2.20E-16	-0.6812

[a] Linear regression between RBP expression and the *mean/SD/CD* expression of all its target genes. Spearman correlation coefficient and P values for analysis are shown.

Supplementary Table 2: The details of the used data.

Experiment	Accession	SRA_file	Pubmed
SRR014	SRR014335	SRR014335.sra	pubmed(19096707)
SRR014	SRR014336	SRR014336.sra	pubmed(19096707)
SRR014	SRR014337	SRR014337.sra	pubmed(19096707)
SRR014	SRR014338	SRR014338.sra	pubmed(19096707)
SRR014	SRR014339	SRR014339.sra	pubmed(19096707)
SRR014	SRR014340	SRR014340.sra	pubmed(19096707)
SRR014	SRR014341	SRR014341.sra	pubmed(19096707)
SRR014	SRR014342	SRR014342.sra	pubmed(19096707)
SRR014	SRR014343	SRR014343.sra	pubmed(19096707)
SRR014	SRR014344	SRR014344.sra	pubmed(19096707)
SRR014	SRR014345	SRR014345.sra	pubmed(19096707)
SRR014	SRR014346	SRR014346.sra	pubmed(19096707)
SRR014	SRR014347	SRR014347.sra	pubmed(19096707)
SRR014	SRR014348	SRR014348.sra	pubmed(19096707)
SRR014	SRR014349	SRR014349.sra	pubmed(19096707)
SRR014	SRR014350	SRR014350.sra	pubmed(19096707)
SRR039	SRR039228	SRR039228.sra	pubmed(20635087)
SRR039	SRR039229	SRR039229.sra	pubmed(20635087)
SRR039	SRR039177	SRR039177.sra	pubmed(21072241)
SRR039	SRR039178	SRR039178.sra	pubmed(21072241)
SRR039	SRR039179	SRR039179.sra	pubmed(21072241)
SRR039	SRR039180	SRR039180.sra	pubmed(21072241)
SRR070	SRR070667	SRR070667.sra	pubmed(21145465)
SRR089	SRR089718	SRR089718.sra	pubmed(20944018)
SRR089	SRR089720	SRR089720.sra	pubmed(20944018)
SRR089	SRR089721	SRR089721.sra	pubmed(20944018)
SRR089	SRR089723	SRR089723.sra	pubmed(20944018)
SRR089	SRR089724	SRR089724.sra	pubmed(20944018)
SRR089	SRR089725	SRR089725.sra	pubmed(20944018)
SRR089	SRR089726	SRR089726.sra	pubmed(20944018)
SRR089	SRR089727	SRR089727.sra	pubmed(20944018)
SRR089	SRR089728	SRR089728.sra	pubmed(20944018)
SRR089	SRR089729	SRR089729.sra	pubmed(20944018)
SRR089	SRR089732	SRR089732.sra	pubmed(20944018)
SRR089	SRR089734	SRR089734.sra	pubmed(20944018)
SRR089	SRR089735	SRR089735.sra	pubmed(20944018)
SRR089	SRR089736	SRR089736.sra	pubmed(20944018)
SRR089	SRR089737	SRR089737.sra	pubmed(20944018)
SRR089	SRR089738	SRR089738.sra	pubmed(20944018)
SRR089	SRR089739	SRR089739.sra	pubmed(20944018)

SRR089	SRR089740	SRR089740.sra	pubmed(20944018)
SRR089	SRR089741	SRR089741.sra	pubmed(20944018)
SRR089	SRR089742	SRR089742.sra	pubmed(20944018)
SRR089	SRR089743	SRR089743.sra	pubmed(20944018)
SRR089	SRR089744	SRR089744.sra	pubmed(20944018)
SRR089	SRR089745	SRR089745.sra	pubmed(20944018)
SRR089	SRR089746	SRR089746.sra	pubmed(20944018)
SRR089	SRR089747	SRR089747.sra	pubmed(20944018)
SRR122	SRR122033	SRR122033.sra	pubmed(21697827)
SRR122	SRR122107	SRR122107.sra	pubmed(21697827)
SRR122	SRR122141	SRR122141.sra	pubmed(21697827)
SRR122	SRR122142	SRR122142.sra	pubmed(21697827)
SRR122	SRR122143	SRR122143.sra	pubmed(21697827)
SRR122	SRR122149	SRR122149.sra	pubmed(21697827)
SRR122	SRR122150	SRR122150.sra	pubmed(21697827)
SRR122	SRR122155	SRR122155.sra	pubmed(21697827)
SRR122	SRR122176	SRR122176.sra	pubmed(21697827)
SRR122	SRR122177	SRR122177.sra	pubmed(21697827)
SRR122	SRR122186	SRR122186.sra	pubmed(21697827)
SRR122	SRR122187	SRR122187.sra	pubmed(21697827)
SRR122	SRR122188	SRR122188.sra	pubmed(21697827)
SRR122	SRR122198	SRR122198.sra	pubmed(21697827)
SRR122	SRR122199	SRR122199.sra	pubmed(21697827)
SRR122	SRR122200	SRR122200.sra	pubmed(21697827)
SRR122	SRR122201	SRR122201.sra	pubmed(21697827)
SRR122	SRR122202	SRR122202.sra	pubmed(21697827)
SRR122	SRR122203	SRR122203.sra	pubmed(21697827)
SRR122	SRR122204	SRR122204.sra	pubmed(21697827)
SRR122	SRR122205	SRR122205.sra	pubmed(21697827)
SRR122	SRR122206	SRR122206.sra	pubmed(21697827)
SRR122	SRR122207	SRR122207.sra	pubmed(21697827)
SRR122	SRR122208	SRR122208.sra	pubmed(21697827)
SRR122	SRR122209	SRR122209.sra	pubmed(21697827)
SRR122	SRR122210	SRR122210.sra	pubmed(21697827)
SRR122	SRR122211	SRR122211.sra	pubmed(21697827)
SRR202	SRR202029	SRR202029.sra	pubmed(21730963)
SRR202	SRR202030	SRR202030.sra	pubmed(21730963)
SRR202	SRR202031	SRR202031.sra	pubmed(21730963)
SRR202	SRR202032	SRR202032.sra	pubmed(21730963)
SRR202	SRR202033	SRR202033.sra	pubmed(21730963)
SRR202	SRR202034	SRR202034.sra	pubmed(21730963)
SRR202	SRR202035	SRR202035.sra	pubmed(21730963)
SRR202	SRR202036	SRR202036.sra	pubmed(21730963)

SRR202	SRR202037	SRR202037.sra	pubmed(21730963)
SRR202	SRR202038	SRR202038.sra	pubmed(21730963)
SRR202	SRR202039	SRR202039.sra	pubmed(21730963)
SRR202	SRR202040	SRR202040.sra	pubmed(21730963)
SRR202	SRR202041	SRR202041.sra	pubmed(21730963)
SRR202	SRR202042	SRR202042.sra	pubmed(21730963)
SRR202	SRR202043	SRR202043.sra	pubmed(21730963)
SRR202	SRR202044	SRR202044.sra	pubmed(21730963)
SRR309	SRR309119	SRR309119.sra	pubmed(21072241)
SRR309	SRR309120	SRR309120.sra	pubmed(21072241)
SRR332	SRR332049	SRR332049.sra	pubmed(21921191)
SRR332	SRR332050	SRR332050.sra	pubmed(21921191)
SRR332	SRR332051	SRR332051.sra	pubmed(21921191)
SRR332	SRR332052	SRR332052.sra	pubmed(21921191)
SRR363	SRR363968	SRR363968.sra	pubmed(22341442)
SRR363	SRR363969	SRR363969.sra	pubmed(22341442)
SRR363	SRR363970	SRR363970.sra	pubmed(22341442)
SRR363	SRR363971	SRR363971.sra	pubmed(22341442)
SRR387	SRR387838	SRR387838.sra	pubmed(22194413)
SRR387	SRR387839	SRR387839.sra	pubmed(22194413)
SRR387	SRR387840	SRR387840.sra	pubmed(22194413)
SRR387	SRR387841	SRR387841.sra	pubmed(22194413)
SRR387	SRR387842	SRR387842.sra	pubmed(22194413)
SRR387	SRR387843	SRR387843.sra	pubmed(22194413)
SRR387	SRR387844	SRR387844.sra	pubmed(22194413)
SRR387	SRR387845	SRR387845.sra	pubmed(22194413)
SRR387	SRR387846	SRR387846.sra	pubmed(22194413)
SRR387	SRR387847	SRR387847.sra	pubmed(22194413)
SRR387	SRR387848	SRR387848.sra	pubmed(22194413)
SRR387	SRR387849	SRR387849.sra	pubmed(22194413)
SRR387	SRR387850	SRR387850.sra	pubmed(22194413)
SRR387	SRR387851	SRR387851.sra	pubmed(22194413)
SRR387	SRR387852	SRR387852.sra	pubmed(22194413)
SRR387	SRR387853	SRR387853.sra	pubmed(22194413)
SRR387	SRR387854	SRR387854.sra	pubmed(22194413)
SRR387	SRR387855	SRR387855.sra	pubmed(22194413)
SRR387	SRR387856	SRR387856.sra	pubmed(22194413)
SRR387	SRR387857	SRR387857.sra	pubmed(22194413)
SRR387	SRR387858	SRR387858.sra	pubmed(22194413)
SRR387	SRR387859	SRR387859.sra	pubmed(22194413)
SRR387	SRR387860	SRR387860.sra	pubmed(22194413)
SRR387	SRR387861	SRR387861.sra	pubmed(22194413)

SRR387	SRR387862	SRR387862.sra	pubmed(22194413)
SRR387	SRR387863	SRR387863.sra	pubmed(22194413)
SRR387	SRR387864	SRR387864.sra	pubmed(22194413)
SRR387	SRR387865	SRR387865.sra	pubmed(22194413)
SRR387	SRR387866	SRR387866.sra	pubmed(22194413)
SRR387	SRR387867	SRR387867.sra	pubmed(22194413)
SRR387	SRR387868	SRR387868.sra	pubmed(22194413)
SRR387	SRR387869	SRR387869.sra	pubmed(22194413)
SRR387	SRR387870	SRR387870.sra	pubmed(22194413)
SRR390	SRR390921	SRR390921.sra	pubmed(22177264)
SRR390	SRR390922	SRR390922.sra	pubmed(22177264)
SRR390	SRR390923	SRR390923.sra	pubmed(22177264)
SRR390	SRR390924	SRR390924.sra	pubmed(22177264)
SRR390	SRR390925	SRR390925.sra	pubmed(22177264)
SRR390	SRR390926	SRR390926.sra	pubmed(22177264)
SRR390	SRR390927	SRR390927.sra	pubmed(22177264)
SRR390	SRR390928	SRR390928.sra	pubmed(22177264)
SRR390	SRR390929	SRR390929.sra	pubmed(22177264)
SRR390	SRR390930	SRR390930.sra	pubmed(22177264)
SRR390	SRR390931	SRR390931.sra	pubmed(22177264)
SRR390	SRR390932	SRR390932.sra	pubmed(22177264)
SRR390	SRR390933	SRR390933.sra	pubmed(22177264)
SRR390	SRR390934	SRR390934.sra	pubmed(22177264)

Supplementary Figures

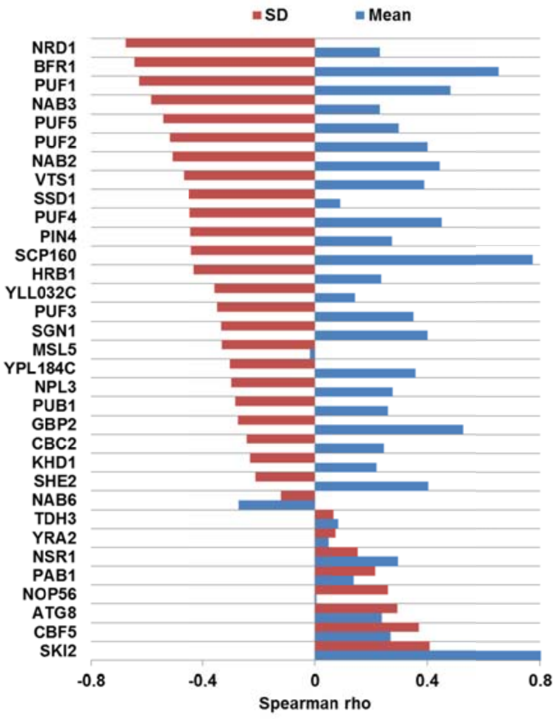

Supplementary Figure 1. The correlation coefficients (spearman rho) between 33 RBP gene expression and the mean (and SD) of their target gene expression. RBPs are ordered based on the spearman rho for the SD values.

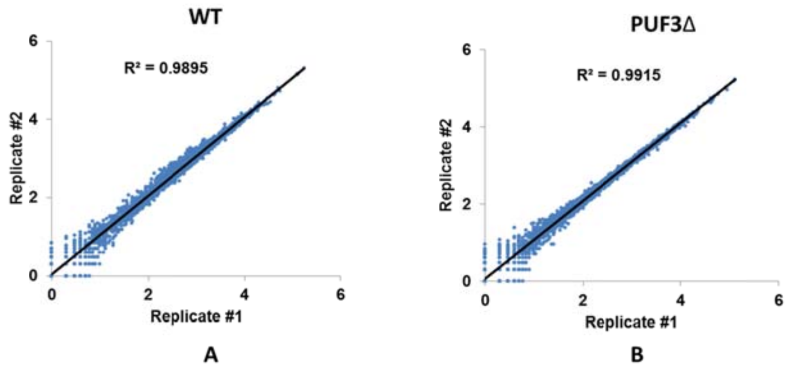

Supplementary Figure 2. The RNA-Seq results for the WT parental strain (**A**) and the PUF3Δ strain (**B**) in YPGE. The correlations of sequence reads between two replicates ($long_{10}$RPKM) are shown.
